# University of New York

**Published:** 1877-06-20

**Authors:** 


University of New York.—See the advertisement of this old and well-established Medical College, in present issue. It needs no commendation, being widely known as ranking high among the very first on the continent.

